# PS-07 - A UKHSA FIRST: EMBEDDING STUDENT NURSES IN HEALTH PROTECTION PRACTICE WITH NORTHUMBRIA UNIVERSITY

**DOI:** 10.1093/pubmed/fdag046.056

**Published:** 2026-07-09

**Authors:** Mrs Aneliese Bickle, Simone Thorn Heathcock, Suzanne Clark, Laura Gray, James Wade, Edward Stevenson, Chelsea Gordon

**Affiliations:** UK Health Security Agency; UK Health Security Agency; UK Health Security Agency; UK Health Security Agency; UK Health Security Agency; UK Health Security Agency; UK Health Security Agency

## Abstract

**Background:**

In 2025, the North East Health Protection Team (NE HPT), in partnership with Northumbria University and UKHSA’s Professional Standards and Development team, delivered UKHSA’s first-ever student nurse placement in Health Protection. The pilot aimed to address workforce sustainability challenges, raise awareness of Health Protection careers, and strengthen early-career exposure to public health practice.

**Objectives:**

This evaluation assessed whether the pilot met its aims, examining impacts on student learning, staff development, operational capacity, and feasibility for wider national rollout.

**Methods:**

A mixed-methods approach included surveys of students, Practice Assessors and Supervisors within the NE HPT, and implementation group members; analysis of student contributions and NMC proficiencies; a joint UKHSA–Northumbria University debrief; and a cost-benefit analysis exploring operational and strategic value.

**Results:**

Students reported an exceptionally positive experience, describing the NE HPT as the most supportive placement of their training. They developed confidence in outbreak management, enquiry handling, case follow-up and multidisciplinary working, achieving a broad range of NMC proficiencies. Staff noted positive cultural impact and meaningful contributions to routine operational flow, though some experienced supervision pressures during winter demand and concurrent registrar onboarding. Implementation group feedback praised strong partnership working but identified challenges around contracting, onboarding delays and security processes. The cost-benefit analysis demonstrated substantial operational and strategic value for the NE HPT, with moderate activity levels bringing the pilot close to full cost recovery and higher student involvement generating a clear net financial gain - firmly positioning the model as a high-return investment for UKHSA.

**Conclusions:**

The NE HPT pilot demonstrated that student nurse placements in Health Protection are feasible, impactful and strategically beneficial. As UKHSA’s inaugural nursing placement, the model provides a strong foundation for national expansion and future workforce strengthening.

